# Cell of Origin and Genetic Alterations in the Pathogenesis of Multiple Myeloma

**DOI:** 10.3389/fimmu.2019.01121

**Published:** 2019-05-21

**Authors:** Benjamin G. Barwick, Vikas A. Gupta, Paula M. Vertino, Lawrence H. Boise

**Affiliations:** ^1^Department of Hematology and Medical Oncology, Emory University School of Medicine, Atlanta, GA, United States; ^2^Winship Cancer Institute, Emory University, Atlanta, GA, United States; ^3^Department of Biomedical Genetics and the Wilmot Cancer Institute, University of Rochester Medical Center, Rochester, NY, United States

**Keywords:** multiple myeloma, MGUS, plasma cell, B cell, genetics, epigenetics, IgH translocations, MYC

## Abstract

B cell activation and differentiation yields plasma cells with high affinity antibodies to a given antigen in a time-frame that allows for host protection. Although the end product is most commonly humoral immunity, the rapid proliferation and somatic mutation of the B cell receptor also results in oncogenic mutations that cause B cell malignancies including plasma cell neoplasms such as multiple myeloma. Myeloma is the second most common hematological malignancy and results in over 100,000 deaths per year worldwide. The genetic alterations that occur in the germinal center, however, are not sufficient to cause myeloma, but rather impart cell proliferation potential on plasma cells, which are normally non-dividing. This pre-malignant state, referred to as monoclonal gammopathy of undetermined significance or MGUS, provides the opportunity for further genetic and epigenetic alterations eventually resulting in a progressive disease that becomes symptomatic. In this review, we will provide a brief history of clonal gammopathies and detail how some of the key discoveries were interwoven with the study of plasma cells. We will also review the genetic and epigenetic alterations discovered over the past 25 years, how these are instrumental to myeloma pathogenesis, and what these events teach us about myeloma and plasma cell biology. These data will be placed in the context of normal B cell development and differentiation and we will discuss how understanding the biology of plasma cells can lead to more effective therapies targeting multiple myeloma.

## A Brief History of Plasma Cells and Malignancy

The study of malignancies that would ultimately be resolved to plasma cells was intertwined with, and necessary for the discovery of plasma cells and their function. Perhaps the first report of the plasma cell malignancy multiple myeloma described as “*mollities ossium*” by Samuel Solly in 1844 characterized two cases of patients who presented with symptoms including fatigue, bone pain, and multiple fractures (1). The author noted, that although rare, these were certainly not the first cases. Upon autopsy it was revealed that the bone marrow of both patients was replaced with a red substance filled with distinctive looking large cells [reviewed by Kyle and Rajkumar (2)]. The second patient noted that his urine stiffened his clothes, and a sample was sent for examination by Dr. Henry Bence Jones who confirmed the semi-solid urine would liquefy upon heating but resume its viscous consistency upon cooling (3, 4). Dr. Bence Jones emphasized the importance of obtaining urine samples for diagnosis, a practice that continues today.

Contemporaneous observations in immunity would lay a foundation for understanding the cellular source of these neoplasms and the Bence Jones proteins. The seminal work of John Fewster and Edward Jenner in smallpox demonstrated acquired immunity, which Jenner would later use to successfully protect patients through inoculation with cowpox (5). This led to discoveries in 1890, where Emil von Behring and Kitasato Shibasaburo showed that the serum of animals immunized with sub-lethal doses of dipetheria and tetanus contained an antitoxin (6). This proved the existence of an adaptive humoral immune system. The following year Paul Ehrlich described this antitoxin component as an “antibody” and in his 1908 Nobel laureate speech predicted the existence of cells that recognize these toxins using a “toxin receptor” and amazingly anticipated that “the antitoxin is nothing else but discharged components of the cell, namely receptors discharged in excess” (7). Although the term “plasma cell” had already been coined (8), it would be more than 40 years before the cellular source of this immunity was discovered.

Several more cases of *mollities osseum* were reported and in 1873 J. von Rustizky coined the phrase multiple myeloma (9). In 1900 James H. Wright concluded that the cells prevalent in multiple myeloma “are essentially plasma cells, or immediate descendants of them” (10). However, this did not explain the presence of proteinurea or Bence Jones proteins. In 1947, plasma cell formation was correlated with antibody production implicating plasma cells as the cellular source of antibodies (11). Korngold and Lipari determined in 1956 that multiple myeloma patients often had “electrophoretically homogeneous” Bence Jones proteins (12), which would later be shown to be identical to protein in the serum of the same patients (13). These monoclonal proteins corresponded to one of the two immunoglobulin light chains that were named kappa and lambda after Korngold and Lipari. Later the delineation of T and B lymphocytes (14) [reviewed by Max Cooper (15)] would lead to the identification of B cells as the precursors to plasma cells.

Advances in electrophoresis and the invention of the immunoblot allowed for more routine testing of immunoglobulin proteins in the serum and urine. In 1961, Jan Waldenström described a monoclonal band in patients with hypergammaglobulinemia many of whom had multiple myeloma or macroglobulinemia, but other patients had no symptoms of malignancy (16). Importantly, Waldenström delineated monoclonal proteins as indicative of neoplasm or a pre-malignant disease (now known as monoclonal gammopathy of undetermined significance or **MGUS**). This was in contrast to polyclonal proteins that were indicative of an inflammatory response.

Today, the cellular and molecular etiology of multiple myeloma as well as the programming of normal B cell development and plasma cell differentiation have been elucidated to a great extent. Like their discoveries, we have learned much about multiple myeloma from studying the normal processes of plasma cell differentiation and *vice versa*. Despite the incredible progress made and knowledge gained, over 130,000 new cases of multiple myeloma occur every year worldwide (17), including over 30,000 cases in the US alone (18). It is now known that myeloma is a progressive disease preceded by an asymptomatic stage called MGUS (19, 20) often followed by an intermediate stage referred to as smoldering multiple myeloma (**SMM**), prior to symptomatic newly diagnosed multiple myeloma (**NDMM**), and finally relapsed and/or refractory multiple myeloma (**RRMM**). Despite the incredible progress made, it is still very difficult to identify MGUS patients who will progress from those whose condition will remain benign. This is a major problem as MGUS is present in 3% of the population over 50 years of age, and progresses to multiple myeloma at a rate of ~1% per annum (21, 22). There is now a formidable arsenal of therapies for multiple myeloma, and thus far the most successful agents are targeted at plasma cell biology, which is largely retained by multiple myeloma (23). While most patients benefit from these treatments, ultimately and unfortunately, most still succumb to disease resulting in almost 100,000 deaths per year worldwide (17).

## B Cell Development, Plasma Cell Differentiation, and Myelomagenesis

B cell development, much like plasma cell neoplasms, progresses through a series of well-defined stages. Current data suggest that a distinguishing attribute of plasma cell malignancies is the differentiation state at which the transformation manifests. This defining characteristic can be exploited to better identify vulnerabilities of multiple myeloma through the study of non-malignant B cells and plasma cells (23). A comprehensive description of these processes has been provided for both B cell development (24, 25) and plasma cell differentiation (26–29), and is beyond the scope of this current review. However, a brief description of these processes is essential to understanding the mechanistic underpinnings and etiology of myelomagenesis.

Like all immune cells, B cells are derived from hematopoietic stem cells that primarily develop in the bone marrow (30, 31) or fetal liver (32). Hematopoietic stem cells can successively differentiate into multi-potent progenitors, common lymphoid progenitors, and eventually mature B cells through the stages pre-pro-B, pro-B, pre-B, immature B, and transitional B cells. In the mouse, this process requires the transcription factors including E2A (33), PU.1 (34), and PAX5 (35) as well as interleukin 7 (IL7) cytokine signaling (36). It is important to point out there are key differences in human B cell development (37, 38), which is not dependent upon IL7 (39). However, in both mice and humans the recombination activated genes, RAG1 and RAG2, physically recombine the variable (V), diversity (D), and joining (J) segments of the immunoglobulin genes (40, 41). Mechanistically, RAG proteins work by recognizing and excising recombination signal sequences, which are conserved heptamer and nonamer sequences separated by a spacer (42). This proceeds first at the immunoglobulin heavy chain (**IgH**) D → J segments (pre-pro-B), and then V → DJ segments (pro-B). If a productive (in-frame) IgH gene is recombined, it is then transcribed, translated, and expressed on the surface with a surrogate light chain (composed of VPREB and IGLL1), which triggers light chain recombination at the V → J segments (light chains contain no diversity segments) marking the pre-B stage. This occurs first at the kappa light chain and if no productive allele is made, then at the lambda light chain. Surface expression of the paired heavy and light chains—referred to as the B-cell receptor (**BCR**)—marks the immature B cells stage, after which B cells can transition from the bone-marrow into the periphery and secondary lymphoid tissues where they mature.

**Figure 1:**
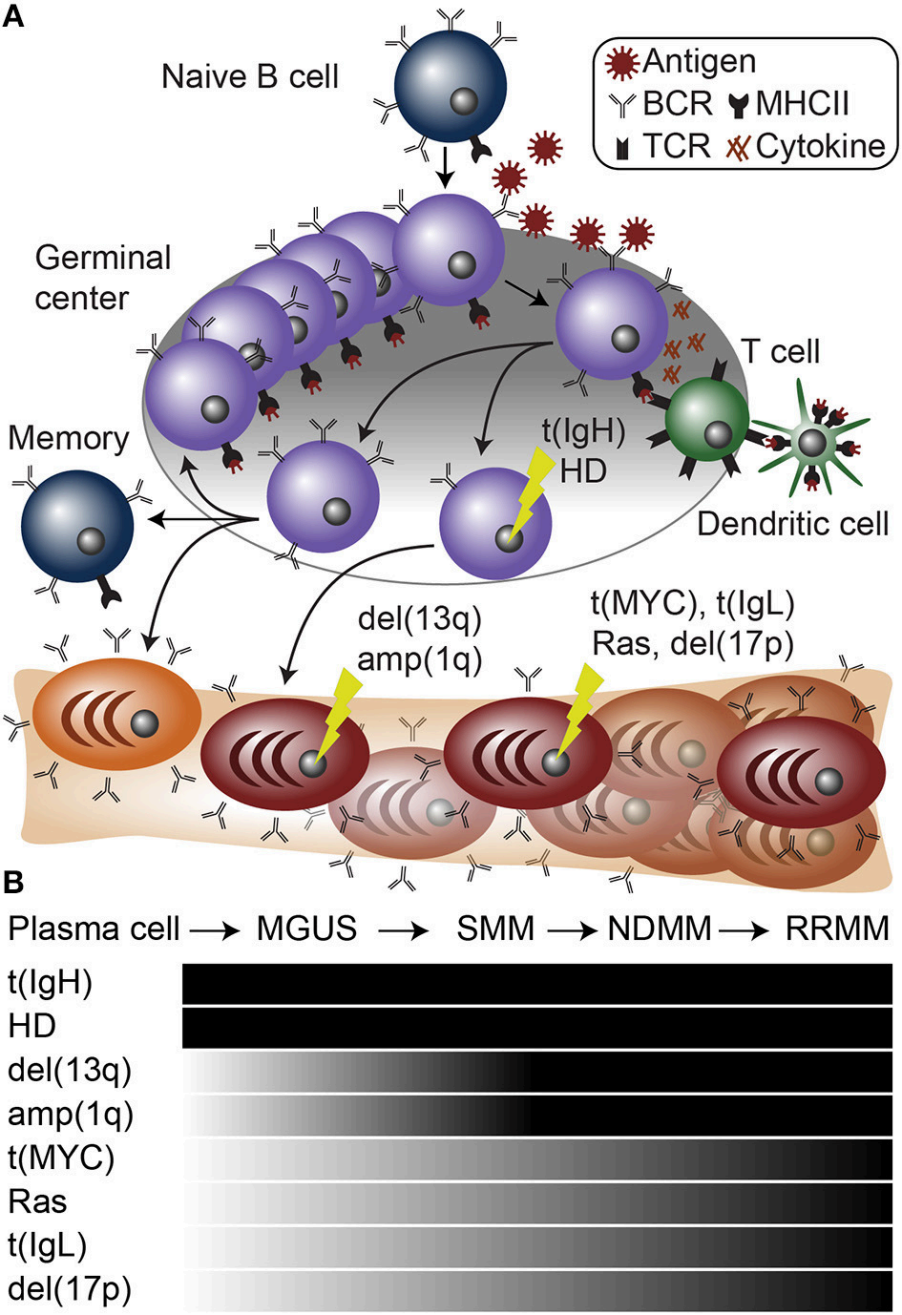
Plasma cell differentiation and myelomagenesis. **(A)** Schematic of B cell differentiation, plasma cell development, and myelomagenesis. Lightning bolts represent genetic mutations common in myeloma. **(B)** Diagram of stages of myeloma progressing from a normal plasma cell to monoclonal gammopathy of undetermined significance (MGUS), smoldering multiple myeloma (SMM), newly diagnosed multiple myeloma (NDMM), and relapse/refractory multiple myeloma (RRMM). Below are common mutations in myeloma and the stages at which they appear.

Mature naïve B cells are mitotically (43) and transcriptionally quiescent (44, 45), but surveil the environment for pathogens which are recognized by toll-like receptors (**TLR**) (46) and the BCR. B cell activation that occurs without cytokine help from T cells, referred to as T-cell independent activation, generally results in acute and shorter lived B cell and plasmablast responses. In contrast, antigens that invoke T cell-dependent (**TD**) cytokine stimulation induce a more complex B-cell activation that results in selection of B cells with higher-affinity B-cell receptors and longer lasting immunity. However, this process is prone to genomic errors that contribute to oncogenesis. Indeed, current data suggests that almost all of myeloma is initiated by mutations associated with TD responses. TD B-cell activation requires BCR-mediated endocytosis of protein antigens, which are subsequently degraded and ectopically presented by the major histocompatibility complex class II (**MHC-II**) (47). When an antigen peptide presented by MHC-II on a B cell is recognized by a cognate T cell receptor (**TCR**), this induces an immunological synapse and T cell stimulation. This causes T cell expression of CD40 ligand (CD40L) (48) that induces B cell CD40 signaling (49), as well as polar release of T cell cytokines IL4 (50, 51), IL21, and IL6 (52) resulting in potent B cell activation. In particular, IL6 not only induces B cell activation, but is a potent growth stimulant for plasma cells and myeloma (53). This stimulation induces rapid B cell proliferation, which forms a lymphoid structure called a germinal center [Figure 1A; reviewed in (54, 55)]. During the germinal center reaction, B cells continuously cycle through rounds of division and selection for high-affinity antibodies, which are made through two types of somatic alterations termed somatic hypermutation (**SHM**) (56) and class-switch recombination (**CSR**), both of which are mediated by the activation-induced cytidine deaminase (**AID**) (57). AID deaminates cytosines on single-stranded DNA resulting in mutations of the immunoglobulin heavy and light chains or SHM. SHM of the heavy and light chains has the potential to increase antibody-antigen affinity through mutation of the complementarity determining region. This results in more efficient antigen uptake and presentation, resulting in more T cell stimulation and selection of B cell clones with high-affinity antibodies to a given antigen. CSR occurs when IgH somatically recombines the constant region μ and its splice isoform δ with one of the alternative constant regions γ3, γ1, α1, γ2, γ4, ε, or α2. CSR occurs via AID-dependent recombination of switch regions located just 5' of each constant region resulting in recombination of a new IgH constant region (58–60). This process requires DSBs, and can result in aberrant recombination with other genomic regions causing translocations. Indeed, there is now substantial evidence that myeloma initiating alterations are a result of errors in CSR.

## Primary Genetic Events in Gammopathies

**Figure 2:**
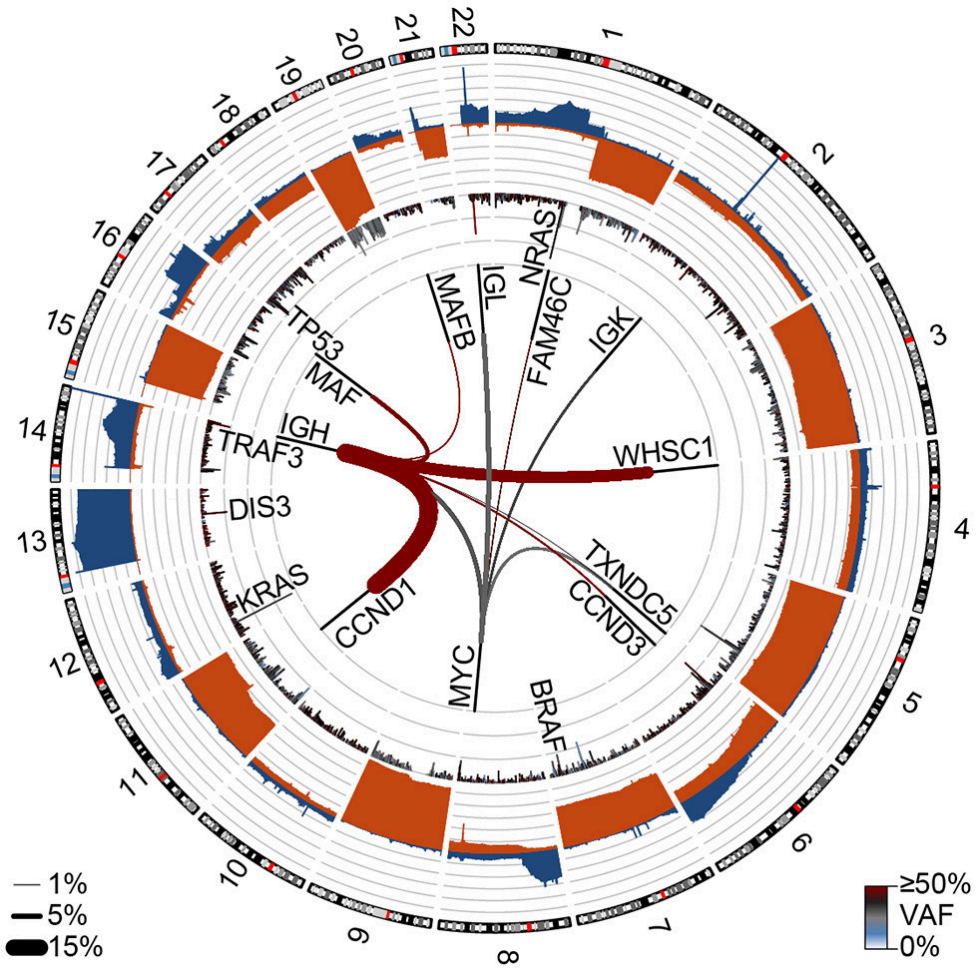
Genetic events in newly diagnosed multiple myeloma (NDMM). Circos plot showing copy number losses (blue) and gains (orange) in the outer ban (gray lines indicate 10% of the population). Mutations are shown on the inner ban, where the frequency of non-synonymous mutations and the variant allele frequency (VAF) are shown for 500 kb regions. Translocations are shown on the inside where the frequency is denoted by line thickness (key bottom left) and color denotes the VAF. Data are from 850 NDMM patients part of the MMRF CoMMpass study (dbGaP phs000748.v7.p4).

A dichotomy of genetic aberrations accounts for the large majority, if not all of myeloma initiating events. First, approximately half of myeloma cases contain an aneuploidy of several odd numbered chromosomes including 3, 5, 7, 9, 11, 15, 19, and 21. This is referred to as hyperdiploidy (**HD**), and will be further discussed below. The second type of founding genetic event is almost mutually exclusive with hyperdiploid myeloma and involves translocations of the IgH locus (61) (Figure 1B). IgH translocations juxtapose the IgH enhancers to one of a half dozen oncogenes including any of the three Cyclin D genes (CCND1-3), WHSC1 (also known as NSD2 or MMSET), MAF, or MAFB [reviewed in (62–64)] (Figure 2). When present, these translocations are clonal alterations (i.e., present in all tumor cells) in all stages of MGUS or myeloma and emanate from the IgH constant chain switch regions implicating them as errors in CSR that occurred during B cell activation in the germinal center (65, 66). Consistent with this, more than 90% of myelomas express class-switched IgH constant chains and almost all display SHM identifying them as post-germinal center cells.

### Cyclin D Dysregulation

Cyclin D dysregulation is the most common type of IgH translocation, which involve t(11;14), t(12;14), and t(6;14) translocations that juxtapose the IgH enhancer(s) with CCND1 (15–20% of NDMM), CCND2 (~1%), and CCND3 (1–4%), respectively (65, 67, 68). All three Cyclin D genes function by activating CDK4 and CDK6 that in turn phosphorylate and inactivate RB allowing for E2F activation and cell cycle progression (63). Although these translocations result in aberrant expression of their respective Cyclin D genes, overexpression of at least one Cyclin D gene appears to be an early and unifying event in plasma cell malignancies (69). For instance, IgH translocations to MAF or MAFB result in high levels of CCND2 (70); IgH-WHSC1 translocations result in moderate levels of CCND2, and hyperdiploid disease results in overexpression of CCND1 (located on chromosome 11) or expression of both CCND1 and CCND2 (69). Conversely, CCND3 expression is less frequent and seems to be primarily a result of t(6;14) (69). Although most Cyclin D translocations occur at the switch region breakpoints, a subset of t(11;14) CCND1 translocations originate from the V(D)J region, suggesting that they may be the result of errors in V(D)J recombination during B cell development (71). Earlier work indicated myeloma-specific idiotypes reacted with some pre-B cells from the same patient, suggesting some myeloma may have origins in B cell development (72). However, these early studies were limited to two cases and it is unclear if pre-B cells with a myeloma idiotype harbor the genetic mutations that result in malignancy. Furthermore, should these errors occur during B cell development, it is not clear what causes these to manifest in myeloma rather than mantle cell lymphoma, which routinely have t(11;14) translocations originating from the V(D)J region (73). Regardless, it is clear that these translocations result in aberrant CCND1 expression, which predisposes to malignancy.

### IgH-WHSC1 or t(4;14)

IgH-WHSC1 or t(4;14) are the second most common translocation, occurring in ~15% of NDMM, and in most cases results in the dual dysregulation of both WHSC1 and FGFR3 (74, 75). These are mostly reciprocal translocations that occur almost exclusively at the IgM switch region and split WHSC1 and FGFR3 on the telomeric side of chromosome 4p. This often results in IgH-WHSC1 fusion transcripts and/or loss of the 5′ exons of WHSC1 (76). Subsequently, the IgH Eμ enhancer drives expression of WHSC1 on the derivative of chromosome 4, while the IgH 3′ enhancers drive expression of FGFR3 on the derivative of chromosome 14 (62, 74). For some time it was unclear whether FGFR3 or WHSC1 was the definitive oncogenic factor, however, ~25% of t(4;14) myelomas do not have the reciprocal FGFR3 translocation and lack FGFR3 expression (76, 77). This suggests that WHSC1 is the essential transforming element, although FGFR3 overexpression and activating mutations likely contribute to pathogenesis. It is also possible that FGFR3 expression is required for MGUS initiation but is subsequently lost in a subset of cases. WHSC1 is now known to be a histone 3 lysine 36 (H3K36) methyltransferase that catalyzes di-methylation of histone 3 lysine 36 (H3K36me2) (78, 79). Pervasive H3K36me2 in t(4;14) myeloma is associated with accessible chromatin and gene dysregulation (79). However, how WHSC1 results in myelomagenesis or CCND2 upregulation is not well understood and difficult to trace due to the genome-wide effects H3K36me2.

### IgH-MAF and IgH-MAFB

IgH-MAF and IgH-MAFB translocations are the least common class of primary IgH translocation, and result from t(14;16) and t(14;20), respectively (80, 81). These are present in approximately 5–10% of NDMM cases, with MAF being more common than MAFB (66). MAF induces expression of CCND2 through a MAF binding motif in the CCND2 promoter, as well as Integrin B7 leading to increased adhesion to bone marrow stromal cells (70). In addition to upregulating MAF, t(14;16) translocation breakpoints disrupt WWOX, a tumor suppressor gene in breast and prostate cancers, where it is also commonly deleted (82, 83). The contribution of WWOX to t(14;16) myeloma is still unclear as there is little to no evidence of biallelic inactivation and most research has focused on the oncogenic effects of MAF (62).

### Hyperdiploidy

Hyperdiploidy is the other common type of initiating genetic event in plasma cell malignancies. Hyperdiploidy is almost mutually exclusive with IgH translocations, and hyperdiploid myeloma tend to have a better prognosis than IgH-translocated multiple myeloma (61, 66, 84). Unlike IgH translocations, it is very difficult to trace the oncogenic effects of hyperdiploidy to a causative element(s) due to the aneuploidy of numerous chromosomes. Compounding the difficulty of pinpointing the pathogenic elements in hyperdiploid myeloma, model systems for hyperdiploid myeloma are lacking. For instance, of the roughly 80 multiple myeloma cell lines, more than 90% have IgH translocations and there are no commonly used hyperdiploid cell lines (62). The good prognosis of hyperdiploid myeloma and lack of cell line models suggests that hyperdiploidy rarely results in extramedullary disease or plasma cell leukemia as most cell lines are derived from patients with disease that is independent of the bone marrow microenvironment (85). Hyperdiploidy is also hypothesized to occur during rapid germinal center proliferation that results in chromosome segregation errors. However, it is not clear if this is one catastrophic event or a series of sequential errors that must occur prior to a clonal outgrowth.

Both IgH translocations and hyperdiploid myeloma are found to be clonal at all stages of gammopathy, which is consistent with them being founding genetic events (Figure 1B). Despite these large genomic changes, almost all myeloma has multiple genetic events present upon diagnosis, suggesting that primary events initiate MGUS, but are not sufficient to cause symptomatic disease.

## Secondary Genetic Events in Gammopathies

In addition to the primary genetic events described above, presentation of myeloma is regularly accompanied by several other major chromosome abnormalities including deletion of chromosome 13q [del(13q)], amplification of chromosome 1q [amp(1q)], and deletion of chromosome 1p [del(1p)] (Figure 1). All three of these alterations involve regions tens of megabase in size and thus similar to hyperdiploidy, pinpointing the causative element(s) is difficult. However, contributing elements are speculated for all of these aberrations with varying degrees of supportive data.

### Del(13q)

Del(13q) occurs in almost 50% of NDMM (86) and is found to be clonal, but is less frequent in MGUS where it is either sub-clonal or clonal (87). Del(13q) co-occurs with t(4;14) and t(14;16) myeloma and was once considered to be marker of poor prognosis, but this outcome appears to have been overcome by the use of proteasome inhibitors (88). Generally, the entire arm of 13q is lost, and contains several loci that may contribute to myeloma pathogenesis. Notably RB1, which prevents cell cycle progression by sequestering E2F transcription factors, is located on 13q14. However, 13q loss is primarily mono-allelic and rarely are there mutations or deletions that result in biallelic RB1 inactivation (64). In contrast to RB1, the exosome endoribonuclease DIS3 is mutated in ~10% of NDMM and ~75% of these mutations occur in del(13q) myeloma suggesting biallelic loss of DIS3 occurs in most DIS3 mutated myeloma (66, 89, 90). Finally, 13q14 is also deleted in Chronic Lymphocytic Leukemia (CLL), albeit in a more punctate fashion, allowing researchers to pinpoint the DLEU2/miR-15a/16-1 locus as a minimally deleted region. Deletion of this region causes a lymphoproliferative disease in mice (91). A similar analysis independently identified the same locus as a minimally deleted region in myeloma (92), but it remains to be determined whether DLEU2, miR-15a, or miR-16-1 have tumor suppressor function in myeloma.

### Amp(1q)

Amp(1q) occurs in 40% of patients and is associated with worse prognosis (93, 94). The poor prognosis appears to be dose-dependent as patients with 4 or more copies of chromosome 1q do worse than those with three (95). These additional copies of 1q likely have a proportional effect on expression of 1q genes, as a gene signature of high-risk myeloma is composed of a large number of 1q genes (96, 97). Putative oncogenes may include CKS1B, which facilitates ubiquitinylation and degradation of the cyclin dependent kinase inhibitor CDKN1B (p27^KIP1^) (98). Approximately two-thirds of amp(1q) coincide with del(13q), which is a significant co-occurrence between the two events (90, 99). If CKS1B and RB1 are the myeloma-inducing genetic alterations on amp(1q) and del(13q), respectively, questions remain as to why two alterations are needed in the same pathway in addition to overexpression of a Cyclin D gene. This might be explained by the somewhat rare nature of cell cycle progression in myeloma where <1% of cells are actively synthesizing DNA (64). Alternatively, it may be a polygenic effect or other genes may be responsible for the deleterious effects of these alterations. One such gene on chromosome 1q is MCL1, a BCL2-family anti-apoptotic protein that is induced during plasma cell differentiation and essential for plasma cell and myeloma cell survival (100–102). There are now MCL1 inhibitors in early phase clinical trials, and it will be important to understand if these are more effective against myeloma with amp(1q) that overexpresses MCL1 (103–105), as discussed further below.

### Del(1p)

Del(1p) occurs in 20–25% of patients and often co-occurs with hypodiploidy (loss of chromosomes). Unlike hyperdiploidy, hypodiploidy is associated with worse prognosis (106, 107) as is del(1p) (94). The region lost on 1p often includes the cyclin dependent kinase inhibitor CDKN2C, and similar to amp(1q), two-thirds of del(1p) also coincides with del(13q) and mono-allelic loss of RB1. Another promising candidate myeloma suppressor gene located on chromosome 1p, is FAM46C, a non-canonical poly(A) polymerase (108, 109). Inactivating mutations in FAM46C result in a cell survival advantage whereas overexpression causes an unfolded protein response and cell death (110). In addition to being lost in ~25% of NDMM by del(1p), FAM46C is also mutated in ~10% and translocated in ~2.5% of NDMM, supporting its role as a *bona fide* tumor suppressor in multiple myeloma (66, 89, 90).

## Genetic Events of Progression in MGUS and Myeloma

### MYC Structural Variants

MYC structural variants are pervasive in B cell malignancies and myeloma is no exception. MYC structural variants are sometimes present in MGUS, present in ~35% of NDMM, and even more common in RRMM and myeloma cell lines (66, 111). This suggests that MYC alterations promote disease progression. This is further supported by a mouse model of myeloma, in which AID-induced MYC expression only results in myelomagenesis in mouse strains prone to MGUS (112, 113). This suggests that MYC cannot initiate MGUS, but facilitates MGUS progression to myeloma. Consistent with this, IgH-MYC [t(8;14)] translocations are distinct from other IgH translocations in that they are found at sub-clonal levels in NDMM and have extragenic IgH breakpoints (66, 112). Such MYC alterations in myeloma are distinct from other B cell malignancies such as Burkitt lymphomas, where immunoglobulin-MYC translocations are a near universal primary event and IgH-MYC translocations have breakpoints in the IgH switch regions (114, 115). In myeloma, MYC structural variants are spread across at least two broad regions and serve to amplify or transpose large enhancers to drive MYC expression (66, 112, 116). Interestingly, almost all MYC translocations are also accompanied by copy number alterations, with most showing large duplicated sequences at both translocation breakpoints (66, 117). This appears to be a common phenomenon present at other secondary translocations in myeloma and other cancers, however, it is rare at myeloma primary translocations that originate from the CSR regions (66, 117). This key insight into the mechanistic basis of secondary and complex translocations could be explained by synthesis-dependent strand annealing of DSBs with long single-stranded overhangs. Indeed, AID deaminates cytosines on single stranded DNA and is known to initiate genomic instability at heavily transcribed regions of the genome (118), such as the intragenic regions of PVT1, where MYC translocations commonly occur, as well as at the immunoglobulin enhancers.

### Immunoglobulin Light Chain Kappa (IgK) and Lambda (IgL)

Immunoglobulin light chain kappa (IgK) and lambda (IgL) enhancers are often co-opted in complex secondary translocations that drive oncogene expression. IgL translocations occur in ~10% of MGUS and NDMM, but up to 20% of RRMM or myeloma cell lines, whereas IgK translocations are more rare, occurring in <5% of NDMM (64, 66). This is surprising given that two-thirds of human B cells and myeloma cells express IgK and only one-third express IgL. The higher prevalence of IgL translocations can be explained by B cell ontogeny, where IgK VJ rearrangement deletes the IgK enhancer if a productive IgK product is not made (119). Thus, without an enhancer the IgK region is inert if translocated, and consequently all IgK-translocated myelomas express IgK (66). Conversely, IgL-translocated myelomas are found at the normal ratio of two-thirds IgK expressing to one-third IgL expressing, which indicates that the IgL enhancer is constitutively active, and equally prone to translocation even in IgK-expressing myeloma (66). We recently showed that translocations of the IgL locus, but not the IgK locus, were prognostic of poor outcome (66). This was even true when restricting the analysis to the same translocated oncogene. For instance, approximately 40% of both IgK and IgL translocations occur to MYC, but only patients with IgL-MYC translocations have a poor prognosis, despite similar baseline levels of MYC expression from each translocation (66). This suggests that distinct enhancers are differentially susceptible to therapeutic perturbation and myeloma is not only a disease of oncogenes but also an “enhanceropathy.”

### Deletion of 17p [del(17p)] Including TP53

Deletion of 17p [del(17p)] including TP53 is also a marker of poor outcome as well as of genomic instability. Unlike several other prognostic markers TP53 status as a high-risk marker has not waned in the face of modern therapies that target plasma cell biology (107). Del(17p) is rare in MGUS, present in ~10% of NDMM but present in the majority of plasma cell leukemias and associated with extramedullary disease (66, 107, 120, 121). TP53 mutations also occur in ~5% of NDMM, but are primarily restricted to samples with del(17p), suggesting a step-wise progression where del(17p) predisposes to biallelic loss of TP53 by selection for cells with TP53 mutation. The co-occurrence of TP53 mutations with 17p loss results in exceedingly poor outcomes (95), and provides strong evidence that TP53 is the functional tumor suppressor inactivated by del(17p).

### Aberrant NF-κB Signaling

Aberrant NF-κB signaling results from both inactivating mutations of genes that suppress NF-κB signaling (e.g., TRAF3) as well as aberrant upregulation of genes the promote NF-κB signaling (e.g., MAP3K14) (89). There is a broad mutational spectrum encompassing dozens of genes, mostly mutated at a low frequency, that converge on the non-canonical NF-κB pathway in ~20% of NDMM (122, 123). Non-canonical NF-κB signaling provides a pro-survival signal and growth advantage to myeloma cells (122, 123), but it is possible that it occurs by a variety of mechanisms. For instance, NF-κB was discovered as a transcription factor that regulates kappa light chain expression (124), but is now known to also regulate IgH and human IgL expression (125). This suggests that NF-κB signaling may enhance expression of oncogenes translocated to immunoglobulin enhancers in myeloma.

This non-canonical NF-κB signaling in myeloma is in contrast to other B cell malignancies such as Waldenstrom's macroglobulinemia (lymphoplasmacytic lymphoma), where over 80% of cases harbor activating mutations in MYD88 that result in canonical NF-κB signaling (126, 127). This may impart a more “innate-like” B cell response and explain why Waldenstrom's macroglobulinemia is almost exclusively IgM expressing whereas IgM expressing myeloma is very rare. Indeed, long-term follow-up of MGUS cases, 15% of which are of the IgM isotype, indicate that IgM MGUS patients progress almost exclusively to Waldenstrom's macroglobulinemia or non-Hodgkin's lymphoma whereas class-switched MGUS cases progress to multiple myeloma (128).

### Ras Signaling

Ras signaling is a common alteration in myeloma but rare in MGUS (129). KRAS and NRAS are the two most commonly mutated genes in myeloma, each present in ~20% of NDMM (66, 89, 95). Counterintuitively, 15% of KRAS-mutated patients also have NRAS mutations. Given the subclonal nature of most Ras mutations, it is conceivable that in cases where both KRAS and NRAS are mutated, these occur in distinct and non-overlapping clonal populations. Alternatively, it may suggest that not all Ras mutations uniformly activate MAPK signaling. Indeed, this has been recently confirmed by phosphoproteomics in myeloma cell lines (130). In contrast, FGFR3 mutations appear to be a more potent inducer of MAPK signaling and are mutually exclusive with NRAS and KRAS mutations (130, 131).

## The Molecular Program of Multiple Myeloma

As noted above, translocations and chromosomal aberrations serve to dysregulate oncogenes and tumor suppressor genes and given the broad array of mutations in myeloma, it is not surprising that these result in distinct gene expression subtypes. Over a decade ago Zhan et al. used cDNA microarrays to classify myeloma into 7 gene expression subtypes, which mostly reflected the founding genetic mutations (132). These expression subtypes include two CCND1 subtypes, CD-1 and CD-2, both driven by t(11;14) translocations, but CD-2 tended to express more B-cell like markers such as CD20; the HY subtype corresponded with genetic hyperdiploidy; the MF subtype corresponded with MAF and MAFB translocations; the MS corresponded with WHSC1 translocations (WHSC1 was commonly referred to as MMSET at the time); LB or low bone disease was not well defined by gene expression or discernable baseline genetics; PR represented a proliferative disease with poor outcome (132). These subtypes of myeloma are well conserved, as segregation of myeloma based on translocations and Cyclin D expression (TC subtype) resulted in groups with similar characteristics (69). Another independent study from Europe identified 10 groups based on gene expression, which corresponded with those from Zhan et al. but provided slightly more granularity (133). Furthermore, we recently found that gene expression subtypes from Zhan et al. were largely conserved in yet another independent data set using a newer technology (RNA-seq) (66). Thus, it appears the initiating genetic alterations of myeloma appear to imprint a gene expression program such that myeloma is really several diseases.

The gene expression subtypes described above correlate with primary genetic events in myeloma, but the impact of secondary and tertiary genetic mutations on gene expression are harder to discern. This may be due to the often sub-clonal nature of these alterations, which likely result in their effects on gene expression being diluted out by cells without the alteration when profiled *en masse*. Emerging single-cell technologies may eventually be able to address this difficult problem, which would ideally require simultaneous profiling of DNA and RNA. Early experiments of single-cell RNA-seq have provided intriguing data indicating the inter-sample heterogeneity of myeloma cells was segmented into distinct gene expression programs, whereas those from SMM, MGUS, and plasma cells from healthy donors where more homogenous (134). This suggests that sub-clonal genetic differences may underlie these variations in the gene expression program, and has important implications for myeloma cell plasticity and the ability of current therapies to effectively eradicate all clones.

## Plasma Cell Differentiation and Epigenetic Dysregulation in Multiple Myeloma

**Figure 3:**
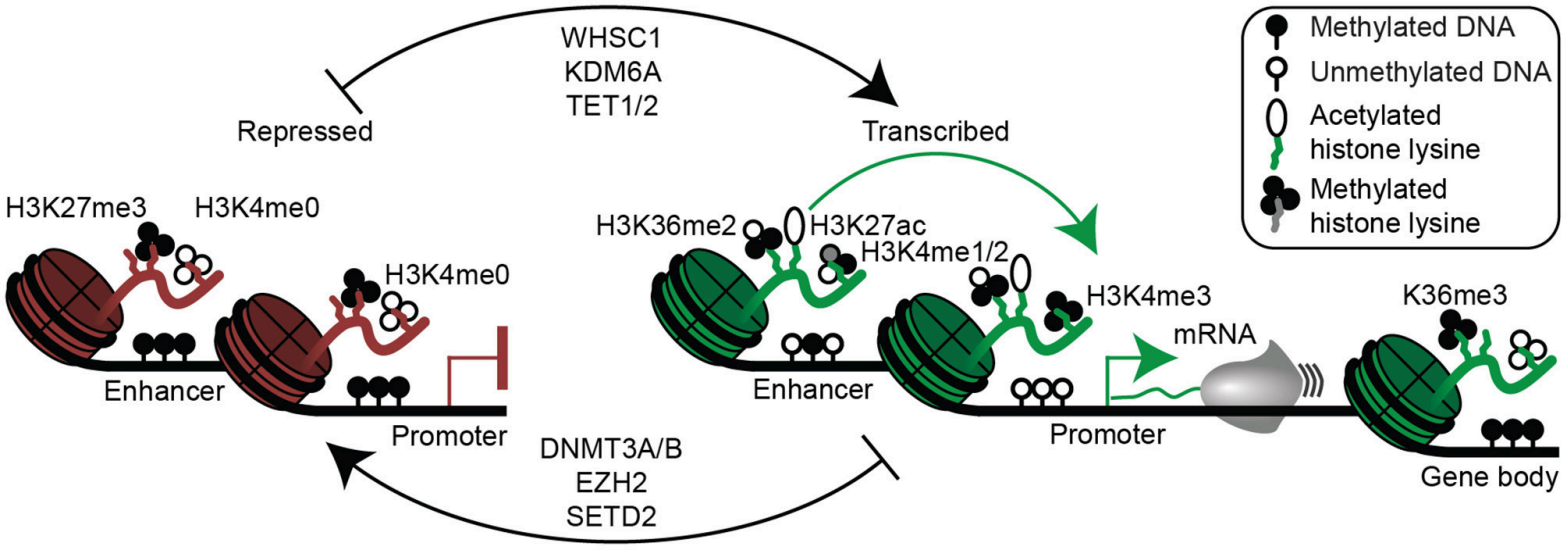
Epigenetic mechanisms of gene regulation in plasma cells and multiple myeloma. Epigenetic modifications associated with gene repression are shown (left) and include histone 3 lysine 27 trimethylation (H3K27me3), lack of histone 3 lysine 4 methylation (H3K4me0), as well as enhancer and/or promoter DNA methylation. Epigenetic modifications permissive to gene transcription are shown (right) and include H3K36me2 (mediated in part by WHSC1), histone 3 lysine 27 acetylation (H3K27ac), H3K4me1/2 at enhancers, H3K4me3 at promoters, and absence of enhancer and promoter DNA methylation. Gene bodies (far right) of actively transcribed genes are often demarcated with H3K36me3 and DNA methylation. Certain enzymes that mediate activating (top) and repressive (bottom) epigenetic modifications relevant to multiple myeloma are shown (middle).

Although the gene expression program appears to be driven by primary genetic alterations, there is clearly a cascade of molecular events that result from these abnormalities. Furthermore, genetic events alone cannot fully explain the gene expression program or phenotype. For instance, the most common translocation in myeloma, t(11;14), is also a defining characteristic of mantle cell lymphoma (73), and t(8;14) translocations that occur as secondary events in myeloma are present as primary events in the majority of Burkitt lymphomas (114, 115). Unlike myeloma, both these lymphomas are believed to originate from pre-germinal center B cell, suggesting that the combination of cancer genetics and cell differentiation state determine the cellular phenotype. Indeed, plasma cell differentiation involves dramatic changes in gene expression, epigenetic reprogramming, and cell morphology (45, 135–140). Thus, genetic alterations may manifest in different phenotypes given their timing in the context of the epigenetic landscape of the cell of origin. These epigenetic changes involve DNA methylation, which primarily occurs on cytosines in CpG dinucleotides (Figure 3). DNA methylation at promoters or enhancers usually functions to repress gene expression by occluding transcription factor binding (141), whereas intragenic DNA methylation corresponds with high levels of gene expression and serves to help prevent transcription from cryptic promoters (142). During germinal center formation and plasma cell differentiation, the histone 3 lysine 27 (H3K27) methyltransferase EZH2 represses plasma cell differentiation genes (e.g., PRDM1, IRF4) by depositing the repressive H3K27me3 histone modification, thereby prolonging the germinal center response (143, 144). The rapid cellular proliferation during the germinal center results in a genome-wide DNA hypomethylation, thereby facilitating activation of plasma cell enhancers, which have the ability to induce plasma cell differentiation (45, 137–139, 145). This explains why activating mutations in EZH2 give rise to germinal center B cell lymphomas (143). These and other epigenetic processes serve to activate plasma cell enhancers and super-enhancers, which are often co-opted to drive oncogene expression (112, 116, 146). Determining the unique trans-acting factors in plasma cell and myeloma cell enhancers may provide an effective way to therapeutically target multiple myeloma (147).

As noted above, one of the most common translocated genes in myeloma is the H3K36 methyltransferase, WHSC1, resulting in a genetic alteration with widespread epigenetic effects. This results in a global increase of H3K36me2 and increased chromatin accessibility as well as a commensurate inhibition of the repressive mark H3K27me3 (78, 79). How this may specifically promote oncogenesis, is still being determined.

In addition to aberrant WHSC1 expression due to t(4;14) translocations, there are a number of mutations in epigenetic enzymes that confer survival advantages to myeloma cells. These include mutations in the H3K27me3 demethylase KDM6A (also known as UTX), where loss of KDM6A function results in increased proliferation, adhesion, and clonogenicity of myeloma cells (148). Unlike the genome-wide effects seen as a result of WHSC1 overexpression, only focal changes on H3K27me3 were observed with KDM6A ablation (148).

Recently, mutations in the isocitrate dehydrogenases, IDH1 and IDH2, have been reported (90). Isocitrate dehydrogenases normally produce α-ketoglutarate, but when mutated result in the accumulation of D-2-hydroxyglutarate, which inhibits Jumonji-C histone demethylases and TET family methylcytosine hydroxylases that require α-ketoglutarate as a co-factor. A consequence of IDH mutations includes altered histone modifications and DNA hypermethylation (149). This may alter the function of transcription factors, such as MYC and MAX, which bind CpG containing E-box elements and are sensitive to DNA methylation state (146, 150). Indeed, we recently showed that loss of function mutations in MAX occur in ~3% of myelomas and alter its binding affinity to methylated and hydroxymethylated E-box transcription factor binding sites (150).

Other modifiers of the DNA methylation pathway mutated in myeloma include TET2 (90), which oxidizes DNA methylation (151) allowing for its removal by base excision repair, as well as the *de novo* DNA methyltransferase DNMT3A (90), which catalyzes DNA methylation at unmethylated CpGs (152). We recently showed that conditional deletion of both *de novo* DNA methyltransferases in B cells results in a loss of DNA methylation at B cell enhancers as well as increased B cell activation and plasma cell differentiation in response to immunization (137). However, the functional impact of these enzymes in myeloma has yet to be elucidated.

In addition to the recent discoveries of mutations in enzymes that regulate DNA methylation, early observations in multiple myeloma showed promoter DNA hypermethylation and gene silencing of the cyclin-dependent kinases inhibitors CDKN2B (p15) and CDKN2A (p16), suggesting they were incapable of preventing cell cycle progression (153). SOCS1, a suppressor of cytokine signaling including the key myeloma cytokine IL6 (53), is also aberrantly silenced by DNA hypermethylation (154, 155). Despite these punctate hypermethylation events, recent genome-wide analyses have found myeloma is mostly characterized by widespread hypomethylation as compared to plasma cells from healthy individuals (156–159). This DNA hypomethylation appears to be progressive as it is more severe in NDMM and RRMM than in MGUS and SMM (156, 159). Indeed, as part of the Multiple Myeloma Research Foundation's CoMMpass project we are performing whole genome bisulfite sequencing on a large cohort of multiple myeloma and have found pervasive hypomethylation organized into megabase domains that are devoid of gene expression. In contrast, DNA methylation was retained in the gene bodies of highly expressed genes. Given the pre-clinical data showing that myeloma cells are sensitive to the DNA methylation inhibitors, such as 5-azacytidine and decitabine (160, 161), the selective sensitivity of multiple myeloma to demethylating agents has yet to be shown *in vivo*.

## Therapeutic Vulnerabilities of Plasma Cells

As our understanding of plasma cell and myeloma biology has improved, so too has our ability to treat myeloma effectively. Like most malignancies diagnosed in the mid twentieth century, myeloma was initially treated with cytotoxic chemotherapy that derived its benefit from attacking any rapidly dividing cell in the body. The alkylating agent melphalan was the first effective treatment for myeloma and in combination with the corticosteroid prednisone formed the backbone of myeloma therapy for 40 years (162, 163). The next major advance came in the 1980's with the introduction of high dose chemotherapy and autologous stem cell rescue, a procedure that is still routinely performed on the majority of eligible myeloma patients today (164, 165). However, it is the addition of novel plasma cell targeted therapy that has had the greatest impact on the improvement in overall outcomes for myeloma patient over the last two decades.

### Proteasome Inhibitors

**Figure 4:**
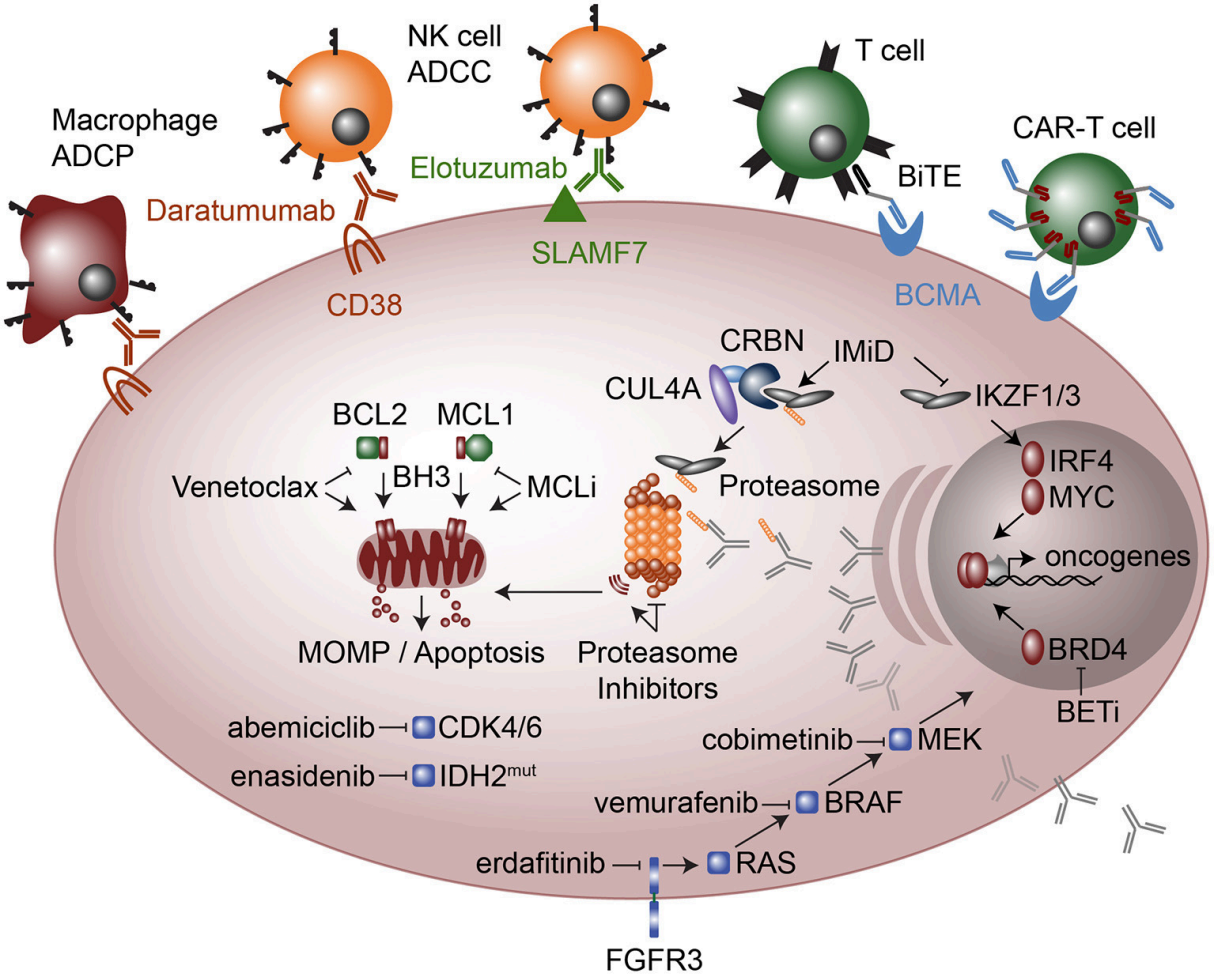
Therapeutic modalities in multiple myeloma. Cellular targeted therapies (top) include chimeric antigen receptor T-cells (CAR-T cells) that target B cell maturation antigen (BCMA) and Bispecific T cell engagers (BiTE), which are two conjugated antibodies, one that recognizes the CD3 receptor on T cells while the other antibody recognizes BCMA. Monoclonal antibodies elotuzumab and daratumumab target SLAMF7 and CD38, respectively and result in myeloma cell killing by Natural Killer (NK) cell mediated antibody-dependent cellular cytotoxicity (ADCC) and in the case of daratumumab also by Macrophage antibody-dependent cellular phagocytosis (ADCP). Molecular modalities include immunomodulatory imide drugs (IMiD; top right) that bind Ikaros (IKZF1) and Aiolos (IKZF3) to Cereblon (CRBN) as part of an E3 ubiquitin ligase complex, which subsequently ubiquitinates IKZF1 and IKZF3 marking them for proteasomal degradation. Proteasome inhibitors (center) result in proteotoxic stress and the unfolded protein response, which plasma cells are particularly sensitive due to their high levels of antibody production. Anti-apoptosis inhibitors (middle left) include MCL1 inhibitors (MCLi) and BCL2 inhibitors such as venetoclax which induce mitochondrial outer membrane permeabilization (MOMP) and apoptosis. Therapeutics targeted at intracellular signaling include the cyclin dependent kinase 4 and 6 (CDK4/6) inhibitor abemiciclib and the mutant IDH2 inhibitor enasidenib. FGFR3 which is highly expressed in most t(4;14) myeloma and sometimes has activating mutations, is targeted with erdafitinib. FGFR3 feeds into RAS / MEK / MAPK signaling, which is targeted with drugs against BRAF (vemurafenib) and MEK (cobimetinib). Finally, a new class of drugs that target transcriptional activators such as bromodomain and extra-terminal (BET) inhibitors the block or degrade BRD4 are being used to target the enhancer machinery present at large enhancers that are often translocated in myeloma such as those found at the immunoglobulin loci.

Proteasome inhibitors target the ability of both normal and malignant plasma cells to produce thousands of antibodies per second. In order to sustain such rapid levels of protein production, the cells rely heavily upon a number of quality control pathways for survival, and it is these pathways that have proven to be an Achilles heel for myeloma. In all cells, protein synthesis occasionally results in the production of misfolded and non-functional peptides that must be quickly disposed of to prevent their accumulation. These peptides are tagged with ubiquitin, which targets them for degradation by the proteasome system. Given the marked protein synthesis activity in myeloma cells, the amount of misfolded protein is similarly amplified, making myeloma even more dependent on the proteasome (166). Proteasome inhibitors block the degradation of misfolded proteins, allowing them to accumulate and ultimately induce cell death through the unfolded protein response (Figure 4). Although the proteasome plays an outsized role in myeloma cells by controlling the unfolded protein response, it has a number of other functions including regulation of signaling pathways, cell-cycle, and DNA repair. Proteasome inhibitors may therefore contribute to cell death through multiple mechanisms. There are currently three proteasome inhibitors approved for myeloma, bortezomib (167), carfilzomib (168), and ixazomib (169). These agents are often combined with dexamethasone, a corticosteroid with anti-lymphocyte activity, and an immunomodulatory drug, particularly during induction therapy (170), but also during maintenance (171) and relapse (172, 173).

### Immunomodulatory Imide Drugs (IMiDs)

Immunomodulatory imide drugs (IMiDs) include thalidomide, the notorious anti-nausea medicine developed in Europe during the 1950s. Despite extensive testing in animals with no side-effects, thalidomide resulted in severe birth defects and in most cases death. However, discovery of the anti-angiogenic properties of thalidomide (174) led to clinical trials which showed it to be an effective agent in treating myeloma (175). This spurred the development of more potent and less toxic analogs of thalidomide, including lenalidomide (176, 177) and pomalidomide (178), now approved for the treatment of myeloma. Despite their efficacy, the mechanism by which IMiDs exert their effect was only recently discovered. IMiDs alter the target specificity of the CUL4A-DDB1-Cereblon E3 ubiquitin ligase (179), and in myeloma, this leads to the degradation of two key plasma cell transcription factors, Ikaros (IKZF1) and Aiolos (IKZF3) (180, 181) (Figure 4). Importantly, IMiDs bind Cereblon through an interaction at residue 391, which is not conserved in mice (182), explaining why thalidomide had no effect on animal studies originally conducted in the 1950s. IMiD-mediated degradation of IKZF1 and IKZF3 results in myeloma cell growth arrest as well as activation of T cells (180, 181), both of which may contribute to anti-myeloma effects of IMiDs. Through loss of IKZF1 and IKZF3, IMiDs also lead to down regulation of IRF4, another essential plasma cell transcription factor (183). IRF4 in turn regulates the expression of MYC (184), a potent oncogene in numerous lymphoid malignancies.

### MYC Aberrant Expression

MYC aberrant expression occurs in the majority of myeloma cases through amplification, translocation, or transcriptional dysregulation (66, 112). Many MYC translocations result in its juxtaposition to immunoglobulin enhancers where the BET bromodomain protein BRD4 is highly associated. As a result, BRD4 inhibitors and degraders are currently being investigated as a method of downregulating MYC expression and inhibiting myeloma cell proliferation (185–187). IMiDs may also target MYC expression through inhibition of IKZF1- and/or IKZF3-regulated enhancers translocated to MYC (66) (Figure 4).

### Immune-Based Therapies

Monoclonal antibodies against cell surface antigens highly expressed on malignant cells have been an important part of cancer therapy since the introduction of rituximab two decades ago. Like other cells of the immune system, plasma cells express cell surface markers that distinguish them from other cells, many of which continue to be expressed on myeloma. The transmembrane glycoprotein CD38 and the immunoreceptor SLAMF7 are the targets of the two monoclonal antibodies currently approved for the treatment of multiple myeloma, daratumamab (188, 189) and eloztuzumab (190), respectively. Daratumumab is capable of inducing complement dependent cytotoxicity, antibody dependent cellular cytotoxicity (ADCC) by NK cells, and antibody dependent cell phagocytosis (ADCP) by macrophages (191, 192), while elotuzumab acts primarily through ADCC and enhancement of anti-myeloma NK cell activity by crosslinking SLAMF7 on the two cell types (193–195) (Figure 4). Development of biologics that target plasma cells has been limited by the number of plasma cell specific markers, and thus a number of other potential targets on myeloma cells are being studied, including GPRC5D (196) and sulfated HLA-I epitopes (197). BCMA is an important cell survival receptor on plasma cells and is the target of the first generation of myeloma directed chimeric antigen receptor (CAR)-T cells and bi-specific T cell engaging (BiTE) antibodies, which are conjugated antibodies binding both myeloma cells and T cells. A neutralizing antibody against the BCMA ligand APRIL is also being developed, as are monoclonal antibodies that deliver cytotoxic drugs more specifically to the antigen expressing cell, so called antibody drug conjugates (ADCs). ADCs targeting the plasma cell markers CD138, CD74, and CD48 are currently undergoing clinical trials.

### Targeting Tumor Specific Biology

Targeting tumor specific biology has been successfully used to treat CML and a number of solid tumors with common driver mutations, but given the degree of genetic heterogeneity in myeloma, this has been less successful than plasma cell directed therapy. Nonetheless, treatment guided by specific oncogenic events in an individual patient's tumor remains an active area of investigation. As described above, alterations in the Ras-MAPK pathway occur in approximately 40% of patients. Although no direct Ras inhibitors exist, treatment with inhibitors of downstream kinases such as BRAF (vemurafenib) and MEK (trametinib, cobimetinib) have been reported in a small number of cases (198–201). Cobimetinib for Ras and Raf mutated patients is also being incorporated into a larger precision medicine trial known as MyDrug (202). Additional arms of this study include inhibitors of IDH2 (enasidenib), FGFR3 (erdafitinib), and CDK (abemiciclib) (Figure 4).

### BCL2 Family Inhibitors

BCL2 family inhibitors represent a new class of drugs that may have applications in a broad range of malignancies. Pro- and anti-apoptotic members of the BCL2 family exist in a delicate state of balance that regulates the survival of both normal and malignant cells (203, 204). The anti-apoptotic proteins BCL2, BCL2L1 (also known as BCL-xL), and MCL1 bind to and sequester pro-apoptotic proteins BIM, BAX, and BAK, preventing them from activating the apoptotic pathway. As normal cells transform into malignant cells they become even more dependent on the anti-apoptotic proteins for survival, leaving them sensitive to inhibitors of the BCL2 family and providing a potential therapeutic window (205–207). Venetoclax, navitoclax (ABT-263), AZD5991, AMG176, and S63845 induce tumor apoptosis by disrupting the function of BCL2, BCL2L1, and/or MCL1 (103, 104, 208). Venetoclax, a BCL2 specific inhibitor, has been approved for the treatment of chronic lymphocytic leukemia (CLL), which originates from BCL2-dependent B cells (209, 210). In contrast, plasma cells upregulate and become dependent upon MCL1, reducing their dependence upon BCL2 (100, 211). As a consequence, myeloma is primarily dependent on MCL1 and inhibitors of MCL1 have shown promising pre-clinical activity (103, 104). Surprisingly, a subset of myeloma characterized by the t(11;14) translocation is co-dependent on BCL2 and responds to BCL2 inhibition with venetoclax (212–218). Dexamethasone further enhances sensitivity to venetoclax by increasing expression of BIM and its binding to BCL2 (219). The biological basis for the BCL2 co-dependence in t(11;14) myeloma remains a mystery, however gene expression profiling of myeloma patient samples did reveal that t(11;14) were composed of two gene expression groups, CD1 and CD2, where CD2 had increased expression of B cell markers such as CD20, PAX5, and VPREB3, suggesting a possible connection with B cells and BCL2 dependence. The bone marrow microenvironment may also play a role in maintaining plasma cell MCL1 dependence through stromal cell mediated secretion of the plasma cell survival cytokine IL6 (220, 221).

## Summary

Throughout the history of multiple myeloma, we have learned a great deal about normal plasma cells from studying the malignant form and *vice versa*. While tremendous progress in the treatment of myeloma has been made over the past 25 years, due in a large part to therapies targeting plasma cell biology, myeloma remains an incurable disease. This necessitates not only the continued study of plasma cell and myeloma biology, but also the germinal center B cell origins of the disease. Clues of these origins are provided by epidemiological correlations. For instance, patients with Gaucher's disease accumulate lysolipids due to a deficiency in glucocerebrosidase, and are more prone to monoclonal gammopathies (222). This was recently leveraged to identify lysolipids as an antigen driving the gammopathy (223). Similarly, it is realized that MGUS incidence increases with obesity and has a higher prevalence in African Americans and in males (64, 223, 224). It will be important to sort out the genetic vs. environmental factors in each of these cases in hopes of minimizing risk of MGUS development. Likewise, it will be very important to identify factors that influence progression of MGUS to myeloma. Clinical trials testing therapeutic intervention to minimize risk of disease progression in SMM are already underway. However, given that 3% of adults over the age of 50 have MGUS (64), less toxic approaches at early stages of clonal gammopathy are needed to minimize chances of disease. Here, even interventions with small effects may have a large impact on cumulative disease burden. It would also be very valuable to accurately identify cases of MGUS that will develop into myeloma. Given the likelihood that myeloma may never be completely eliminated by preventative approaches, better models of disease will be needed to effectively develop the next generation of therapies. For instance, although we have learned a lot from patient derived cell lines models, these do not provide tractable comparisons of different genetic alterations without confounding background genetics. Although CRISPR has revolutionized gene editing, it has yet to be co-opted to induce myeloma translocations and it is unclear if this is possible. However, it is encouraging that other genetic approaches have been able to induce such translocations in murine B cells (225). Finally, given the dependence of most myeloma on the microenvironment, better *in vivo* models will also be needed. Significant efforts have yielded a mouse model of myeloma driven by AID-dependent MYC expression (113), and a humanized mouse capable of sustaining the human immune system including myeloma (226). These systems will need to be further exploited and expanded to better understand how the different genetic subtypes of myeloma respond to therapy and to delineate microenvironmental interactions and dependencies that can be leveraged to better treat multiple myeloma.

## Data Availability

Figure 2 was generated by analysis from 850 newly diagnosed patients from the MMRF CoMMpass study (dbGAP phs000748.v7.p4).

## Author Contributions

BB and VG wrote the manuscript. LB and PV provided editorial input.

### Conflict of Interest Statement

The authors declare that the research was conducted in the absence of any commercial or financial relationships that could be construed as a potential conflict of interest.
